# Genomic Reconstruction of an Uncultured Hydrothermal Vent Gammaproteobacterial Methanotroph (Family *Methylothermaceae*) Indicates Multiple Adaptations to Oxygen Limitation

**DOI:** 10.3389/fmicb.2015.01425

**Published:** 2015-12-23

**Authors:** Connor T. Skennerton, Lewis M. Ward, Alice Michel, Kyle Metcalfe, Chanel Valiente, Sean Mullin, Ken Y. Chan, Viviana Gradinaru, Victoria J. Orphan

**Affiliations:** ^1^Division of Geological and Planetary Sciences, California Institute of TechnologyPasadena, CA, USA; ^2^Division of Biology and Bioengineering, California Institute of TechnologyPasadena, CA, USA

**Keywords:** methane, nitrate, denitrification, deep sea, thermophile, hydrothermal vent, Lau Basin, methane oxidation

## Abstract

Hydrothermal vents are an important contributor to marine biogeochemistry, producing large volumes of reduced fluids, gasses, and metals and housing unique, productive microbial and animal communities fueled by chemosynthesis. Methane is a common constituent of hydrothermal vent fluid and is frequently consumed at vent sites by methanotrophic bacteria that serve to control escape of this greenhouse gas into the atmosphere. Despite their ecological and geochemical importance, little is known about the ecophysiology of uncultured hydrothermal vent-associated methanotrophic bacteria. Using metagenomic binning techniques, we recovered and analyzed a near-complete genome from a novel gammaproteobacterial methanotroph (B42) associated with a white smoker chimney in the Southern Lau basin. B42 was the dominant methanotroph in the community, at ∼80x coverage, with only four others detected in the metagenome, all on low coverage contigs (7x–12x). Phylogenetic placement of B42 showed it is a member of the *Methylothermaceae*, a family currently represented by only one sequenced genome. Metabolic inferences based on the presence of known pathways in the genome showed that B42 possesses a branched respiratory chain with A- and B-family heme copper oxidases, cytochrome *bd* oxidase and a partial denitrification pathway. These genes could allow B42 to respire over a wide range of oxygen concentrations within the highly dynamic vent environment. Phylogenies of the denitrification genes revealed they are the result of separate horizontal gene transfer from other Proteobacteria and suggest that denitrification is a selective advantage in conditions where extremely low oxygen concentrations require all oxygen to be used for methane activation.

## Introduction

Deep-sea hydrothermal vent systems are a significant contributor to the marine methane cycle, considered both a global source and sink of this potent greenhouse gas. Hydrothermal vent systems are common along mid-ocean ridges, back-arc spreading centers, and other subaqueous divergent plate boundaries (Beaulieu et al., 2013) where concentration of methane in vent fluids is ∼100 times higher than the surrounding ocean waters (Welhan and Craig, 1979, 1983). Vents vary in chemical composition, but plumes are often enriched in H_2_S, CH_4_, H_2_, Fe^2+^, and Mn^2+^, which chemosynthetic microbes utilize as energy resources, and also emit trace elements such as copper, zinc, iron, cobalt, and chromium (Dick et al., 2013; Sylvan et al., 2013). As these reduced compounds mix with the open ocean, they oxidize to form chimney structures, or alternatively disperse throughout the water column to provide an important source of trace elements and nutrients for the broader marine system (Elderfield and Schultz, 1996; Tagliabue et al., 2010). Methane emitted from vents provides a significant source of energy for microbial communities (Dick et al., 2013) and is consumed almost entirely by methanotrophic microbes living both near the seafloor and in the water column (Lesniewski et al., 2012). Despite their importance in the global methane eﬄux, the diversity, distribution, and detailed characterization of methane-oxidizing microorganisms from hydrothermal vent environments have not been significantly characterized.

Methanotrophic bacteria utilize methane to provide cellular carbon and energy through oxidation via methane monooxygenase (MMO). Carbon assimilation proceeds by either the ribulose monophosphate (RuMP) pathway found in Type I methanotrophs, or the serine pathway found in Type II methanotrophs (Trotsenko and Murrell, 2008). To date all vent-associated methanotrophs are type I, consistent with reports that type II methanotrophs are not identified in marine systems (Lidstrom, 1988). Using metagenomic and transcriptome sequencing, as well as 16S rRNA or MMO gene assays (Hirayama et al., 2007; Lesniewski et al., 2012; Dick et al., 2013; Li et al., 2014; Anantharaman et al., 2015), Type I methanotrophs belonging to the *Gammaproteobacteria* have been detected at hydrothermal vent sites such as the Lau and Guayams Basin (Tavormina et al., 2010; Dick et al., 2013; Sylvan et al., 2013), off of the Islands of Japan (Hirayama et al., 2007, 2013, 2014) at the Mid-Okinawa Trough, the Trans-Atlantic Geotraverse (Elsaied et al., 2004), and the Rainbow Vent fields (Nercessian et al., 2005). Though these experiments have provided estimates of the abundance and diversity of aerobic methane-oxidizing organisms at hydrothermal vent sites, complete methanotroph genomes from deep-sea hydrothermal vents have yet to be sequenced and interpreted. Such information can contribute to a more complete understanding of the functional capacity of these chemosynthesizers at hydrothermal vent sites.

The role of aerobic methanotrophs in global nitrogen cycling is also of growing interest. Some methanotrophs have been shown to contribute to nitrogen fixation (Auman et al., 2001) and non-specific oxidation of ammonium by MMO is also known to occur, resulting in inadvertent nitrification by methanotrophs (Bédard and Knowles, 1989; Hommes et al., 2001). This oxidative metabolism produces hydroxylamine, a toxic intermediate that can be converted to nitrite via hydroxylamine oxidoreductase (Campbell et al., 2011). Many aerobic methylotrophs possess pathways for assimilatory nitrate reduction, while dissimilatory nitrate reduction pathways are often absent (Stein and Klotz, 2011).

Recently, additional overlap between the methane and nitrogen cycling has been demonstrated. Denitrifying microbes have been implicated in nitrate reduction coupled to methane oxidation using either the reverse methanogenesis pathway in *Candidatus* Methanoperedens nitroreducens (Haroon et al., 2013), a novel intra-aerobic methanotrophic pathway in *Candidatus* Methylomirabilis oxyfera (Ettwig et al., 2010), as well as denitrification-coupled aerobic methanotrophy in bacteria in dysoxic conditions (Kits et al., 2015). Denitrification-coupled aerobic methanotrophy in dysoxic conditions has been demonstrated in the gammaproteobacterium *Methylomonas denitrificans*, which requires trace amounts of molecular oxygen to activate methane but is capable of using oxidized nitrogen as a terminal electron acceptor (Kits et al., 2015).

In this study, metagenomic sequencing was used to sample the microbial community and associated metabolic potential with a white smoker chimney from the Tu’i Malila vent field in the Lau Basin. From this metagenomic dataset, we successfully reconstructed a draft genome from an aerobic methanotroph that, like *M. denitrificans*, is capable of denitirfication-coupled aerobic methanotrophy.

## Materials and Methods

### Site Description and Sample Collection

The Lau Basin in the western Pacific is a back-arc basin that consists of several ridge segments arranged approximately North-South, aligned subparallel to the convergent Pacific-Australian plate margin to the east. A large (∼15 cm) piece of a white smoker chimney, sample #2044C, was collected during cruise tuim06mv on Dive J2_144 (May 21, 2005) from the Tu’i Malila vent field (176° 34.060′ W, 21°59.350′ S; depth 1876 m), located on the Valu Fa Ridge at the southern end of the Lau Basin in the western Pacific Ocean. Fluids emanating from this white smoker were measured at 260°C, with 10–12°C temperatures measured on the exterior surface of the chimney where #2044C was sampled. Upon recovery shipboard, a sterile chisel and hammer were used to subsample the exterior chimney material, followed by storage at -80°C until genomic DNA extraction in 2015.

Mineralogical analysis of sample #2044C by XRD (B. Harrison personal communication) indicates a composition dominated by sphalerite, anhydrite, chalcopyrite (copper iron sulfide), and galena (lead sulfide) with lower contributions of wurtzite (zinc iron sulfide), barite (barium sulfate), pyrite (iron sulfide), and molybdenum.

### Metagenomic Sequencing, Assembly, Binning and Annotation

DNA from sample #2044C was extracted using the MO BIO PowerSoil^®^ DNA isolation kit, following the manufacturer’s instructions, and sequenced using the Illumina HiSeq2500 platform. Raw metagenomic sequencing reads were assembled using megahit 0.1.2 (Li et al., 2015) using default parameters. Contig binning was performed using emergent self-organizing maps using the tetranucleotide frequencies of contigs greater than 2 kbp in size. Tetranucleotide frequencies for contigs were generated using bioruby-kmer_counter 0.1.2 (https://github.com/wwood/bioruby-kmer_counter) using the default parameters. ESOM maps were generated using the databionics-ESOM package (Ultsch and Moerchen, 2005) using the parameters reported by Dick et al. (2009); genome bins were classified manually on the ESOM map. Extracted genome bins were validated using checkM 0.9.7 (Parks et al., 2015). The B42 draft genome was improved by reassembly using spades 3.5.0 (Bankevich et al., 2012). Reads from contigs of the original assembly of the bin and reads that were found in contigs that linked through paired read information were extracted and used as the input to spades. Manual improvement to correct mis-assemblies and join contigs was performed on the spades scaffolds. Annotation of the B42 genome was performed with RAST (Aziz et al., 2008; Overbeek et al., 2014).

### Phylogenetic Analysis

Gene phylogenies for NirK and NarG were constructed by identifying genes in the IMG database (Markowitz et al., 2014) that contained sequence similarity using the BLASTp algorithm using a cutoff score of 1e^-5^. Up to 500 of the best hits were used to construct each phylogenetic tree. The PmoA tree was constructed by selected sequences that were above a cutoff score of 1e^-105^ against the NCBI NR database. Four additional sequences obtained from the metagenomic assembly were also included in the phylogeny of the PmoA tree. Sequences were aligned with Muscle 3.8.31 (Edgar, 2004), using default parameters. Trees were calculated using RAxML 8.1.7 (Stamatakis, 2014) using the following parameters: -f a -k -x 483735 -p 54927 -N 100 -T 16 -m PROTGAMMAWAG.

### Data Availability

The draft genome sequence is available in the Integrated Microbial Genomes (IMG) database under the accession 2623620619.

## Results and Discussion

### Genome Reconstruction and Phylogeny

**FIGURE 1:**
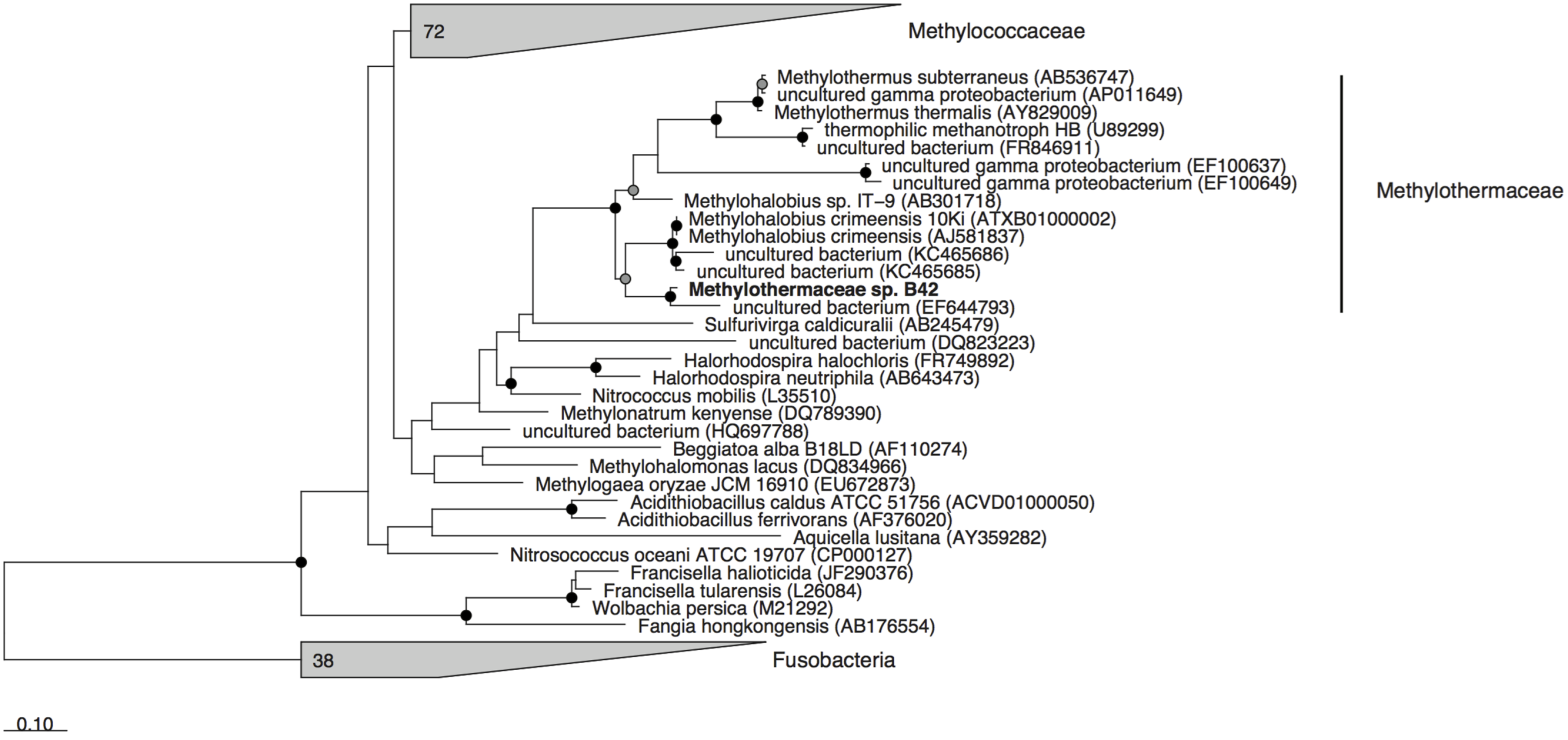
**Maximum likelihood phylogenetic tree of the 16S rRNA gene from cultured *Methanococcales* and associated non-methanotrophic Gammaproteobacteria, and some related environmental clones.** Gray wedges indicate monophyletic groups of sequences. Cultured isolated from the Fusobacteria were used as the outgroup. Nodes with greater than 70 or 90% bootstrap support are indicated with a gray or black circle, respectively. Scale bar indicates substitutions per site.

**FIGURE 2:**
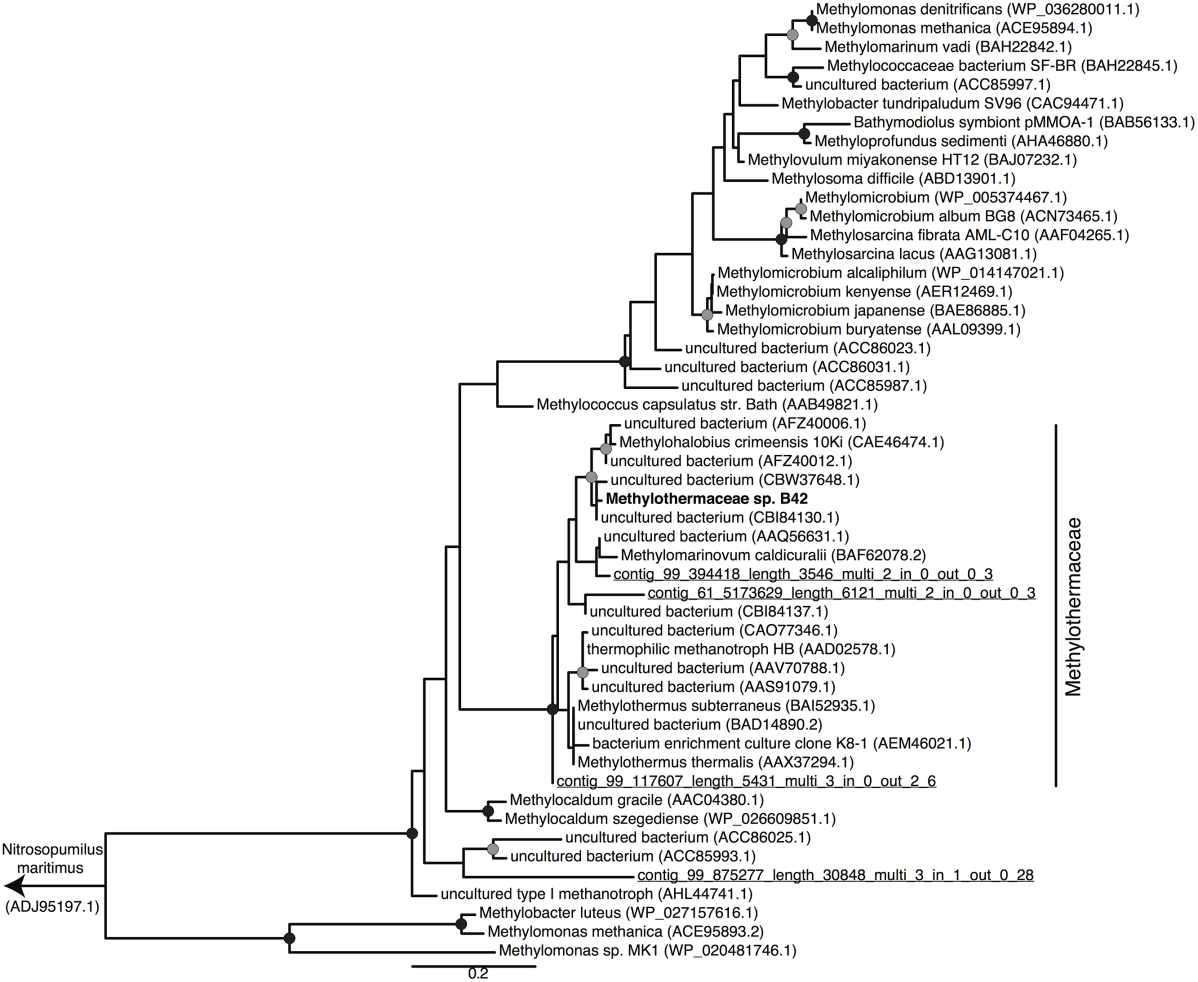
**Maximum likelihood phylogenetic tree of PmoA from cultured *Methanococcales*, and some related environmental clones.** PmoA sequences recovered from the metagenome are underlined and labeled with their contig names. The *Nitrosopumilus maritimus* ammonia monooxygenase subunit A protein was used as the outgroup of the tree. Nodes with greater than 70 or 90% bootstrap support are indicated with a gray or black circle, respectively. Scale bar indicates substitutions per site.

**FIGURE 3:**
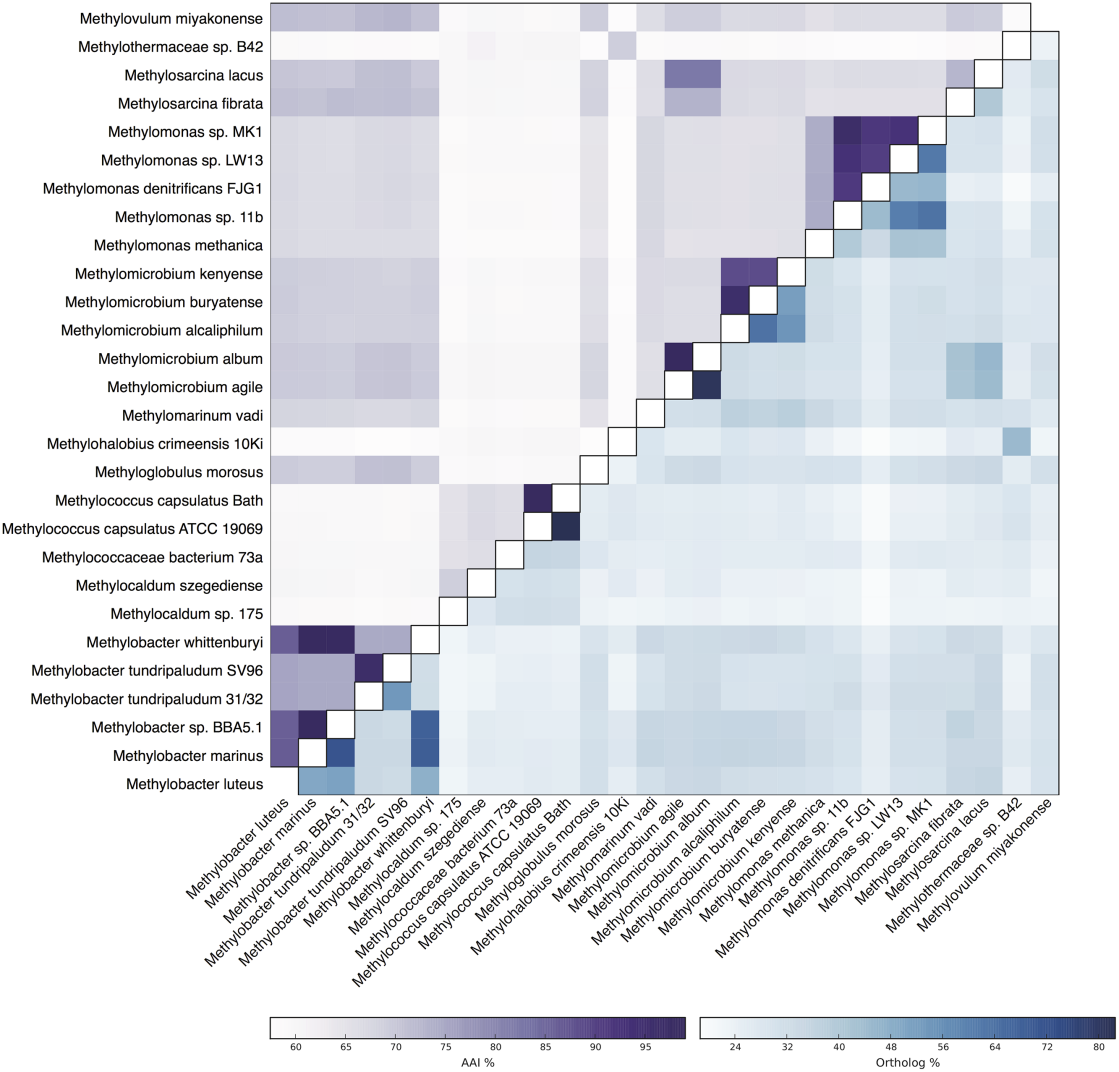
**Heatmap of genome relatedness between B42 and other gammaproteobacterial methanotrophs.** The average amino acid identity (AAI) between homologous proteins between pairs of genomes is shown in the upper triangle. The percentage of total proteins that are homologous is shown in the lower triangle.

Metagenomic sequencing, assembly and binning resulted in a number of high quality genome bins from the hydrothermal vent metagenome. Analysis of the metabolisms of these genome bins identified only a single draft genome that contained the enzymes for aerobic methane oxidation. This genome bin, referred to hereafter as B42, was assembled into 39 scaffolds containing 3.04 Mbp of total sequence at an average coverage of 80x. B42 was estimated to be 97% complete and 1% contaminated based on the presence of single-copy marker genes. The genome bin contained a single complete rRNA operon that allowed for phylogenetic classification as a member of the *Methylothermaceae*, with the closest cultured isolate being *Methylohalobius crimeensis* (**Figure 1**). Phylogenetic placement of the *pmoA* gene is consistent with the 16S rDNA tree indicating phylogenetic affiliation within the *Methylothermaceae* family (**Figure 2**). A search for *pmoA* in the whole metagenome returned four additional copies, three of which were phylogenetically related to the *Methylothermaceae*, while the fourth copy was most closely related to *Methylocaldum* (**Figure 2**). These additional *pmoA* genes were on low coverage contigs (7x – 12x) that indicates that while other methanotrophs are present, B42 is the most abundant and presumably the most important for methane oxidation at this site. The average amino acid identity (AAI) between B42 and all other genome sequences of gammaproteobacterial methylotrophs was below 70%, with the exception of *Methylohalobius crimeensis* (**Figure 3**). This value is lower than the AAI of genomes from other genera such as *Methylobacter* (AAI: 70–95%) or *Methylomonas* (AAI: 75–90%) and suggests that although B42 is most related to *Methylohalobius crimeensis* it may not be a member of the same genus (**Figure 3**).

### Carbon Metabolism

B42 contained all of the necessary RuMP pathway genes (type I methanotroph) for assimilation of methane-derived carbon that has been found in all previously sequenced gammaproteobacterial methanotrophs (Hanson and Hanson, 1996). Notably, this includes all three subunits of the particulate methane monoxygenase (pMMO) that were organized in an operon *pmoCAB*, which is a consistent synteny to other type I methanotrophs (Trotsenko and Murrell, 2008). The B42 genome contained an extra *pmoC* subunit separate from the pMMO operon. Previous hypotheses have suggested that additional *pmoC* genes play a distinct role in the pMMO, possibly to activate pMMO (Stolyar et al., 1999), it may be a neutral duplication, or it may be involved in ammonia oxidation, which is closely related to methane oxidation (Hanson and Hanson, 1996). Since the two copies of *pmoC* are more closely related to each other than to *pmoC* from other *Methylothermaceae*, it likely originated from a recent duplication event. B42 does not appear to possess a homolog of the soluble methane monoxygenase, sMMO, which is found in only some of the gammaproteobacterial methanotrophs (Hanson and Hanson, 1996).

**FIGURE 4:**
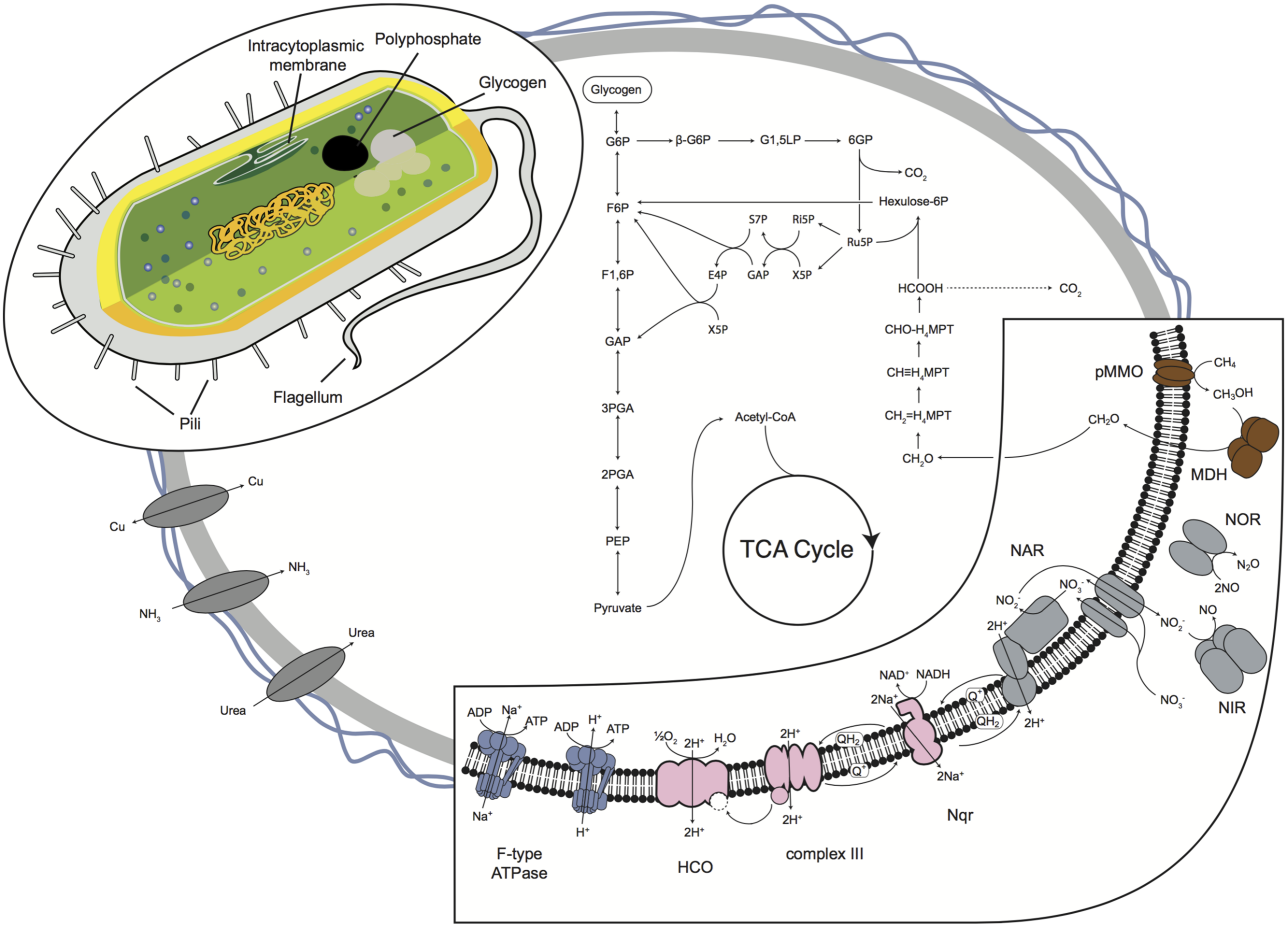
**Model predictions of the central metabolism inferred from the B42 genome sequence.** Important energy generating reactions are shown expanded on the lower left corner. Carbon derived from methane is either completely oxidized to CO_2_ (dashed line) or is assimilated through the RuMP pathway into the glycolysis and TCA cycle. A proposed cellular ultrastructure is shown in the top right. The genome also encodes genes to the construction of pili, flagella and for the accumulation of polyphosphate and glycogen granules. Abbreviations: G6P, glucose 6-phosphate; G1,5LP, 6-phospho D-glucono-1,5-lactone; 6GP, gluconate 6-phosphate; Ru5P, ribulose 5-phosphate; Ri5P, ribose 5-phosphate; X5P, xylulose 5-phosphate; GAP, glyceraldehyde 3-phosphate; S7P, sedoheptulose 7-phosphate; F6P, fructofuranose 6-phosphate; E4P, erythrose 4-phosphate; F1,6P, fructose 1,6-bisphosphate; 3PGA, 3-phospho-D-glycerate; 2PGA, 2-phospho-D-glycerate; PEP, phosphoenolpyruvate; CH_2_ = H_4_MPT, 5,10-methylene-tetrahydromethanopterin; CH_2_≡H_4_MPT, 5,10-methenyltetrahydromethanopterin; CHO-H_4_MPT, 5-formyl-tetrahydromethanopterin.

The pMMO catalyzes the production of methanol, which is further oxidized to formaldehyde by a periplasmic methanol dehydrogenase (MDH; **Figure 4**). MDH transfers electrons through cytochrome cL and cytochrome cH to the terminal heme copper oxidase. B42 utilizes the methenyltetrahydromethanopterin-linked pathway to oxidize formaldehyde to formate. Formate is oxidized to carbon dioxide by NAD-dependent formate dehydrogenase (FDH), with four isoenzymes present in B42 that have high sequence similarity to other members of the *Methylococcaceae*. Formaldehyde can then be assimilated into biomass using the RuMP pathway (**Figure 4**).

B42 has the ability to store excess carbon in the form of glycogen. The genome contains genes involved in glyconeogenesis (glycogen formation) including glycogen synthase, and branching enzymes and glycogen utilizing enzymes such as debranching enzymes, glycogen phosphorylase, and alpha-amylase. Methanotrophs have been observed to use either glycogen or PHB, with a preference for type I methanotrophs to produce glycogen exclusively (Pieja et al., 2011). Interestingly, some cultured members of the *Methylothermaceae* have been observed to contain intracellular granules, reported to be PHB (Heyer et al., 2005; Hirayama et al., 2011, 2014). While there were no genes for PHB production identified in the partial B42 genome, *M. crimeensis* was found to contain some of the genes required for PHB synthesis. Further confirmation of intracellular granules in cultured *Methylothermaceae* is required, however, PHB production may be possible in certain strains of type I methanotrophs.

### Copper Acquisition, Use, and Expulsion

Copper is the metal cofactor for pMMO, and as such, the copper requirement of methanotrophs is estimated to be 10 times that of other organisms (Semrau et al., 2013). B42 appears to lack copper uptake genes for the synthesis of methanobactin; and does not contain the *mmoB* gene, usually associated with the soluble MMO, which acts in copper sensing and regulation (Semrau et al., 2013). However, methanobactin may be confined to a few type II methanotrophs (Kenney and Rosenzweig, 2013), which may mean that B42 uses a novel chalkophore or perhaps the high copper concentrations found at the Tu’i Malila hydrothermal vents (Sylvan et al., 2013) abrogate the need for a copper concentrating mechanism. Copper homeostasis may be achieved in B42 using the cop operon (*copABCD*) that has been shown to be involved in copper import and eﬄux in non-methanotroph gammaproteobacterial model organisms. For example, CopC and CopD have been shown to increase copper uptake in *Pseudomonas* (Cha and Cooksey, 1993), while CopA and CopB are P-type ATPases that can import or export copper across the outer membrane (Cooksey, 1994; Solioz and Stoyanov, 2003).

### Respiration Using a Branched Electron Transport Chain

As an aerobic methanotroph, B42 requires oxygen for the oxidation of methane. Perhaps to survive oxygen limitation, B42 encodes a number of respiratory complexes that allow for respiration over a wide range of oxygen concentrations (**Figure 4**). The B42 genome encodes a complete electron transport chain, including a sodium-translocating NADH:quinone oxidoreductase, Complex III, and genes for utilizing both oxygen and oxidized nitrogen as terminal electron acceptors. These pathways have a variety of evolutionary histories and reflect both vertical inheritance of pathways and horizontal gene transfer.

The genome of B42 contains all of the subunits of two members of the heme-copper oxidase (HCO) superfamily that encode an A-family and a B-family O_2_ reductase. The B-family O_2_ reductase enzymes are adapted to lower concentrations of oxygen than the A-family, having converted a conserved proton channel into an O_2_ channel. This result in a higher affinity for O_2_ but fewer protons pumped per electron (Han et al., 2011). Possessing both A- and B-family HCO genes may allow B42 to respire over a wide range of oxygen concentrations.

B42 also possesses a cytochrome *bd* oxidase, a respiratory quinol:O_2_ oxidoreductase with a very high affinity for O_2_ (Borisov et al., 2011). The *bd* oxidase family is not homologous to the HCO superfamily, and conserves less energy than HCOs as *bd* oxidase donates electrons to O_2_ directly from quinol, bypassing energy conservation at Complex III, and lacks conserved channels for pumping protons (Borisov et al., 2011; Han et al., 2011). However, *bd* oxidase has an extremely high affinity for O_2_, allowing it to be used for respiration at vanishingly low oxygen concentrations, with a Km for O_2_ consumption of 3–8 nM (D’mello et al., 1996; Stolper et al., 2010).

Multiple pathways suggest that B42 is capable of generating or consuming either a H^+^ or Na^+^ gradient for energy. Two separate and complete ATP synthase operons were detected in the genome, one of which was annotated as Na^+^-translocating. Precise understanding of the sequence-level differences between proton and sodium translocating ATP synthases is lacking in most organisms other than *Acetobacterium woodii*, which has a distinct evolutionary history (Müller et al., 2001; Müller and Grüber, 2003). Conclusive evidence for sodium-driven ATP generation in B42 is therefore difficult, however, the closest homolog is found the cultured organism, *M. crimeensis*. Further testing of *M. crimeensis’* membrane kinetics may therefore aid in determining B42′s activity. To generate the Na^+^ gradient, B42 possesses a complete Na^+^-pumping NADH:quinone oxidoreductase operon (*nqrABCDEF*) similar to those described for *Vibrio cholerae* species. These proteins are functionally similar to the proton-translocating protein of the same name, but are evolutionarily distinct, and are composed of non-homologous subunits (Steuber et al., 2014).

B42 may be able to utilize nitrate as an alternative electron acceptor under extreme oxygen limitation (**Figure 4**). As pMMO has an absolute O_2_ requirement to convert methane to methanol, this metabolism could not continue when oxygen is entirely absent. However, denitrification in B42 could be coupled to aerobic methanotrophy under oxygen limitation by utilizing available O_2_ for activating methane with pMMO while using oxidized nitrogen as an electron acceptor, as has been shown recently for *Methylomonas denitrificans* (Kits et al., 2015). Like *M. denitrificans*, B42 appears to contain a cyanoglobin homolog, which in *M. denitrificans* is upregulated under hypoxic conditions and has been hypothesized to bind oxygen for delivery to pMMO (Kits et al., 2015).

**FIGURE 5:**
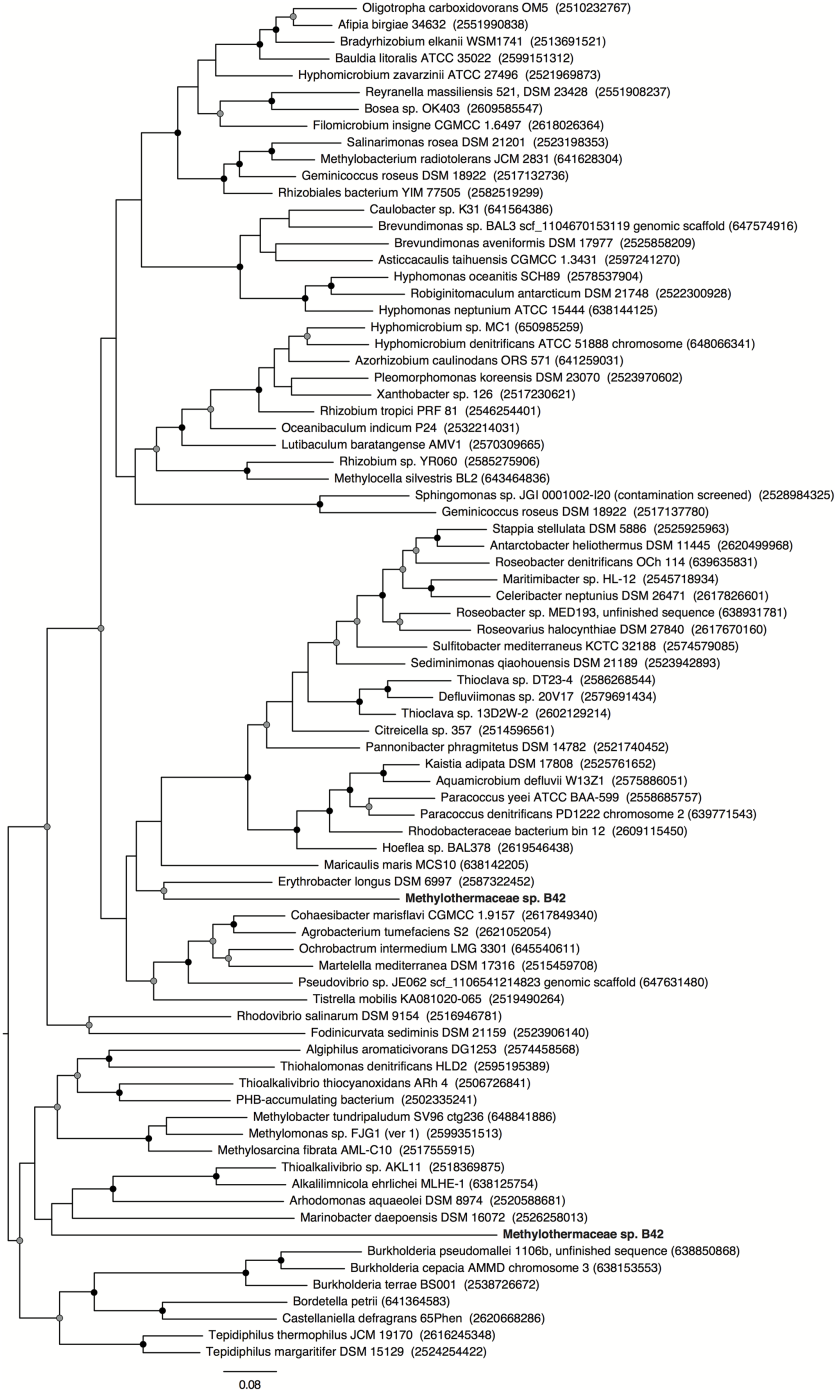
**Maximum likelihood phylogenetic tree of NarG from reference genomes and B42.** Tip labels show the genome name and the IMG gene id in brackets. The tree was rooted at the mid-point node, no outgroup was included. Nodes with greater than 70 or 90% bootstrap support are indicated with a gray or black circle, respectively. Scale bar indicates substitutions per site.

**FIGURE 6:**
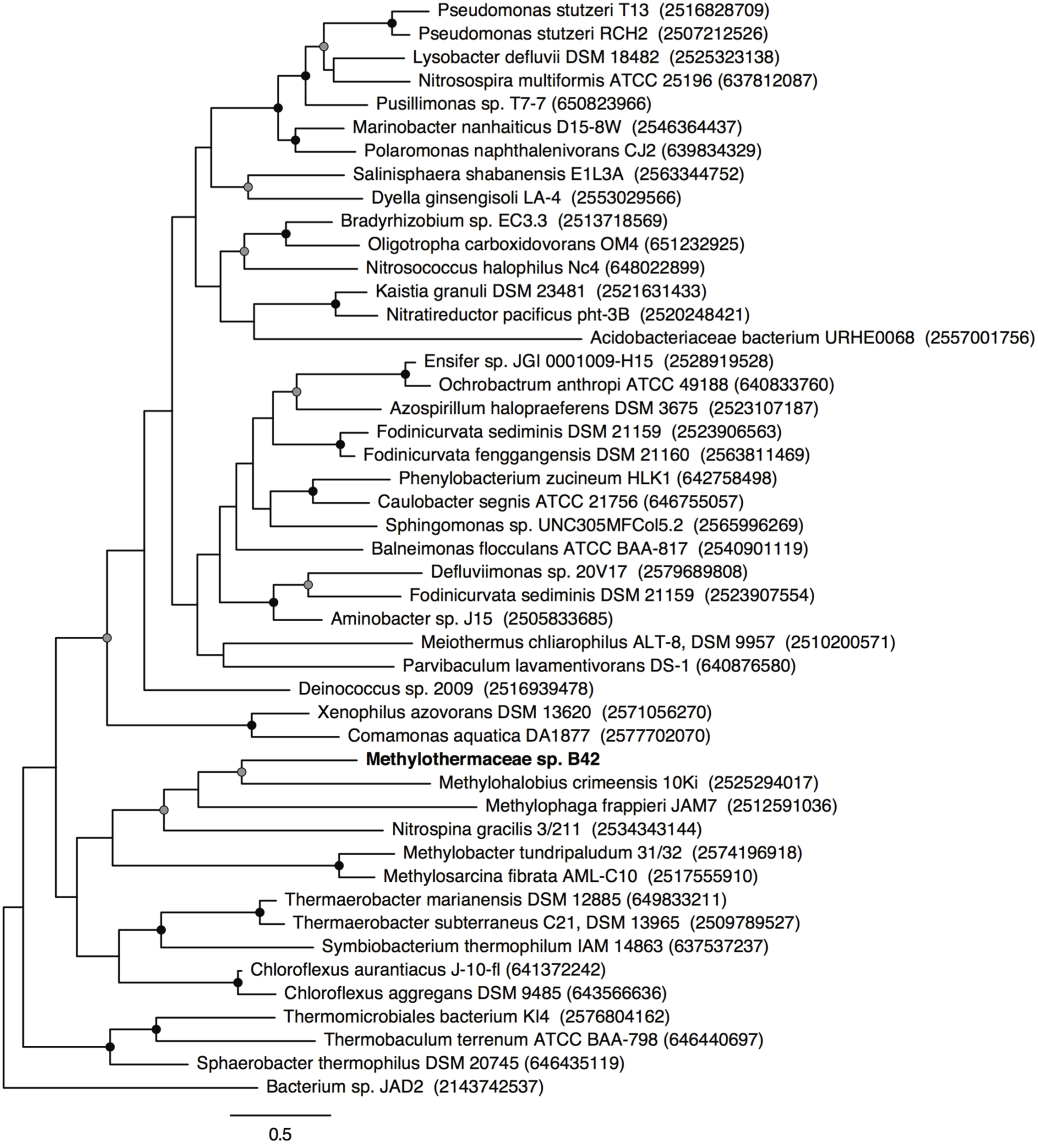
**Maximum likelihood phylogenetic tree of NirK from reference genomes and B42.** Nodes with greater than 70 or 90% bootstrap support are indicated with a gray or black circle, respectively. Scale bar indicates substitutions per site.

An incomplete denitrification pathway is present in the B42 genome to convert nitrate to nitrous oxide (**Figure 4**). Two respiratory nitrate reductase operons (*narGHIJ*) are present and both are adjacent to the nitrate/nitrite transporter (*narK*). Phylogenetic analysis of the NarG gene revealed that the two copies have distinct evolutionary histories (**Figure 5**), suggesting they are the result of independent horizontal transfer events and not due to duplication within the B42 genome. One copy of NarG was most related to sequences from *Alphaproteobacteria*, while the other copy is most related to sequences from the *Gammaproteobacteria* (**Figure 5**). Nitrite reduction occurs using the copper-containing nitrite reductase, *nirK*, and appears to have been inherited from the common ancestor of B42 and *M. crimeensis* (**Figure 6**). Nitrite is a toxic intermediate formed not only from nitrate reduction but also ammonium oxidation. The pMMO enzyme can oxidize small amounts of ammonium, creating hydroxylamine that is converted to nitrite by hydroxylamine dehydrogenase (*hao*), also found in the B42 genome (Nyerges and Stein, 2009). As NirK is found in a number of non-denitrifying members of the *Methylococcaceae*, it may be conserved in methanotrophs, along with hydroxylamine oxidoreductase and nitric oxide reductase, as part of a conserved detoxification pathway for the byproducts of non-specific ammonia oxidation by pMMO. Nitric oxide produced from nitrite reduction can be reduced using the cytochrome c dependent nitric oxide reductase cNOR, a member of the heme copper oxidase superfamily. The final step in denitrification, reduction of nitrous oxide to nitrogen gas, does not appear to be encoded by the genome. Nitrous oxide reduction is often absent from denitrifier genomes, as nitrous oxide is largely non-toxic (Zumft, 1997) and this step conserves less energy, hence when oxidized nitrogen is not limiting it is more energy efficient to allow nitrous oxide to escape (Chen and Strous, 2013).

The complement of terminal electron accepting reactions possessed by B42 implies an organism capable of thriving under a range of oxygen concentrations. While it would conserve the most energy utilizing its A-family HCO under high oxygen conditions, B42 appears to be capable of continuing to respire under low-O_2_ conditions using the B-family HCO or the cytochrome *bd* oxidase, and to be capable of transitioning to incomplete denitrification when oxygen is nearly depleted. Respiratory nitrate reduction by aerobic methanotrophic bacteria has previously been demonstrated in *M. denitrificans* and its presence in the B42 genome may indicate it is also capable of nitrate respiration. Nitrate is generally non-toxic, and its dissimilatory reduction is adaptive as an electron acceptor. The acquisition of denitrification genes could greatly expand the niche of B42 and also suggests an adaptive advantage to using nitrate as an electron acceptor. Fluctuating oxygen concentrations could severely limit growth by aerobic methanotrophs, however, denitrification allows all oxygen to be utilized for activating methane, thus enabling B42 and other denitrifying aerobic methanotrophs to outcompete others for methane in these environments.

It remains unclear how a transition of oxygen to nitrate utilization would be accomplished, and what electron donors would be utilized under denitrifying conditions. An alternative, though perhaps not mutually exclusive hypothesis, is that denitrification and aerobic respiration are run simultaneously. As aerobic respiration and denitrification share many components of the electron transport chain, differing only in the terminal electron acceptor, it has been proposed that a “hybrid” of the two pathways can be run to maximize energy conservation under low-O_2_ conditions and to minimize response times when oxygen levels fluctuate (Chen and Strous, 2013). A final alternative is that B42 utilizes denitrification under anoxic conditions coupled to heterotrophy rather than methanotrophy. Resolution of these possibilities may be possible via isolation of B42 or *in situ* transcriptomic analyses under a range of oxygen concentrations.

## Conclusion

Metagenomic sequencing of a deep-sea hydrothermal vent has recovered the genome of a novel methanotroph, B42, from the family *Methylothermaceae*. B42 was the dominant methanotroph recovered from this white smoker chimney and its genome contained multiple adaptations to varying oxygen concentrations. The presence of denitrification genes has only been identified in three other gammaproteobacterial methanotrophs and suggests that oxygen limitation generates evolutionary pressure for B42 at hydrothermal vents. Coupling of methanotrophy and denitrification suggests that links between the methane and nitrogen cycles are more common than previously recognized. However, the nature of denitrification-coupled methanotrophy is currently unresolved, and requires physiological experiments to determine oxygen concentrations for and dynamics of the transition from oxygen to nitrate as an electron acceptor.

## Author Contributions

VO and VG conceived the initial study of the hydrothermal vent systems. KC performed laboratory work to prepare samples for sequencing. CS performed initial assembly and binning. VO and CS conceived the analysis of the metagenome bin. CS, LW, AM, KM, CV, SM performed analysis of the metagenome bin. CS, LW, AM, KM, CV, SM, VO wrote and revised the manuscript.

## Conflict of Interest Statement

The authors declare that the research was conducted in the absence of any commercial or financial relationships that could be construed as a potential conflict of interest.
